# Supplementary material for: Coxiella burnetii Seroprevalence and Associated Risk Factors in Cattle, Sheep, and Goats in Estonia
Source: Microorganisms. 2023 Mar 23;11(4):819. doi: 10.3390/microorganisms11040819 (PMC10142450; doi:10.3390/microorganisms11040819)
Supplement: Supplementary file 1 [file microorganisms-11-00819-s001.zip › Figure S1.pdf]

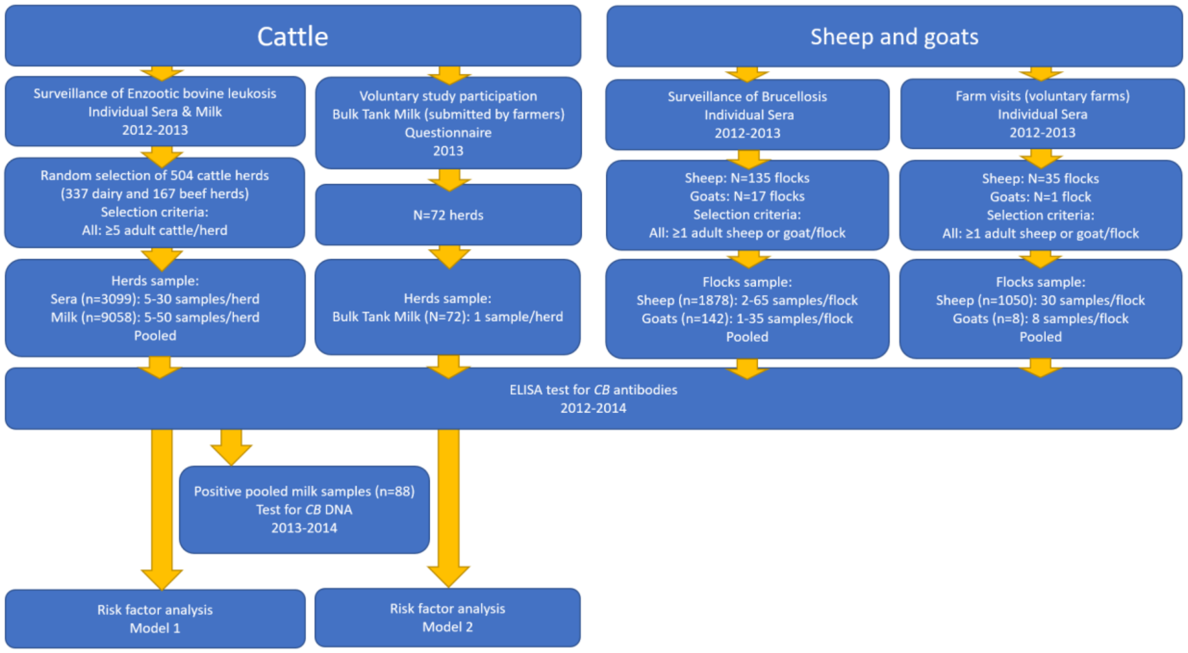

Figure S1. Collection of samples from Estonian cattle, sheep and goats to identify *Coxiella burnetii* seroprevalence and related risk factors
