# Supplementary material for: Coxiella burnetii Seroprevalence and Associated Risk Factors in Cattle, Sheep, and Goats in Estonia
Source: Microorganisms. 2023 Mar 23;11(4):819. doi: 10.3390/microorganisms11040819 (PMC10142450; doi:10.3390/microorganisms11040819)
Supplement: Supplementary file 1 [file microorganisms-11-00819-s001.zip › Table S1.pdf]

Table S1. A questionnaire was used to clarify possible risk factors for *Coxiella burnetii* infection in volunteered dairy cattle herds

1. Are purchased animals quarantined?
   - ☐ Always
   - ☐ Mostly
   - ☐ Sometimes
   - ☐ Mostly not
   - ☐ Never
   - ☐ Not buying new animals

   a. If you answered 'yes', please describe where (in which building and how isolated from other animals) and for how long the animals are quarantined.

   ....

   ....

2. How often do the farm animals participate in animal shows?
   - ☐ Every year
   - ☐ Every second year
   - ☐ Every few years
   - ☐ Even less often
   - ☐ Never

3. Keeping type of animals (mark all correct answers with a cross)

	Tied keeping	Loose keeping	Other (description)
Lactating cows			
Cows/heifers in the final pregnancy stage			
Pregnant heifers			
Young cattle >3 months up to pregnancy			

4. What bedding material is used (mark all correct answers with a cross)?

	Mats	Straw	Peat	Sawdust (chips)	Other (description)
Lactating cows					
Cows/heifers in the final pregnancy stage					
Pregnant heifers					
Young animals >3 months up to pregnancy					
Calves					

5. Grazing of cows

☐ All cows ☐ Only dry cows ☐ No grazing

☐ Other: ....

   a. Grazing of young cattle:

☐ Yes ☐ No ☐ Other: ....

6. If the cattle are grazed

a. Do the pastures come into contact those of other animal owners? ☐ Yes ☐ No

b. Are marshy (wet) areas present on used pastures? ☐ Yes ☐ No

c. Are the pastures in contact with natural waterbodies?

☐ No contact ☐ River/creek ☐ Small lake/pond ☐ Large lake ☐ Sea

d. Do animals drink from natural waterbodies? ☐ Yes ☐ No

e. Are the cattle grazed together with other farmed animals? ☐ Yes ☐ No

f. If the cattle are grazed together with other animals, mark with whom (mark all that apply):

☐ Sheep ☐ Goats ☐ Horses ☐ Other: ……………………………………………………

7. Veterinarian (mark all correct answers with a cross)

☐ The person is a paid employee of the company
☐ A veterinary service is used
☐ The person also serves other companies
☐ Other: ……………………………………………………………………………………………………

8. Inseminator (mark all correct answers with a cross)

☐ The owner inseminates himself/herself
☐ The person is a paid employee of the company
☐ An insemination service is used
☐ The person also serves other companies
☐ Other: ……………………………………………………………………………………………………

9. Is natural insemination/mating with a bull used? ☐ Yes ☐ No

a. If the answer is 'yes', who will be mated? ☐ Heifers ☐ Cows
b. Is the bull borrowed from outside the herd? ☐ Yes ☐ No
c. Is the bull being borrowed from the herd? ☐ Yes ☐ No

10. Do outside workers (including veterinarians, inseminators, and farm equipment maintenance workers) and visitors put on clean protective clothing on the farm?

☐ Always ☐ Mostly ☐ Sometimes ☐ Mostly not ☐ Never

11. Do any of the farm workers keep production animals at home? ☐ Yes ☐ No
