# Supplementary material for: Coxiella burnetii Seroprevalence and Associated Risk Factors in Cattle, Sheep, and Goats in Estonia
Source: Microorganisms. 2023 Mar 23;11(4):819. doi: 10.3390/microorganisms11040819 (PMC10142450; doi:10.3390/microorganisms11040819)
Supplement: Supplementary file 1 [file microorganisms-11-00819-s001.zip › Table S2.pdf]

Table S2. Univariable regression model of risk factors predicting *Coxiella burnetii* seroprevalence in 504 Estonian cattle herds based on surveillance data

Variable	n/N [1]	Prevalence (95% CI [2])	Odds Ratio (95% CI)	p-value
Production type				
Beef cattle herds	12/180	6.67 (3.85–11.29)	1	
Dairy cattle herds	88/324	27.16 (22.61–32.25)	5.22 (2.77–9.85)	<0.001
Herd size				
Small (<101 animals)	30/314	9.55 (6.77–13.31)	1	
Medium (101–300 animals)	30/111	27.03 (19.64–35.95)	3.51 (2.00–6.16)	<0.001
Large (>300 animals)	40/79	50.63 (39.84–61.37)	9.71 (5.44–17.34)	<0.001
No. of animals [3]			1.003 (1.002–1.004)	<0.001
No. of EHF [4]			1.003 (1.002–1.004)	<0.001
No. of ER [5]			1.001 (1.00–1.004)	0.03
No. of Bd'A [6]			1.01 (0.99–1.03)	0.266
No. of Hf [7]			0.99 (0.97–1.01)	0.302
% of beef animals [8]			0.98 (0.97–0.99)	<0.001
Region [9]				
Southwest	14/156	8.97 (5.42–14.50)	1	
Southeast	25/99	25.25 (17.73–34.62)	3.43 (1.68–6.98)	<0.001
Northeast	40/121	33.06 (25.31–41.85)	5.01 (2.57–9.76)	<0.001
Northwest	21/128	16.41 (10.99–23.78)	1.99 (0.96–4.10)	0.061
Season [10]				
Spring (20.03–20.06)	40/222	18.02 (13.52–23.60)	1	
Summer (21.06–21.09)	19/89	21.35 (14.11–30.95)	1.24 (0.66–2.28)	0.499
Autumn (22.09–22.12)	10/59	16.95 (9.48–28.46)	0.93 (0.43–1.99)	0.849
Winter (23.12–19.03)	31/134	23.13 (16.80–30.96)	1.37 (0.81–2.32)	0.243

[1] number of herds with *CB* antibody positive result (n) and number of tested herds (N)
[2] 95% confidence interval (95% CI)
[3] number of registered animals in the herd (No. of animals)
[4] number of animals of Estonian Holstein Friesian breed (dairy breed) in the herd (No. of EHF)
[5] number of animals of Estonian Red cattle breed (dairy breed) in the herd (No. of ER)
[6] number of animals of Blonde d'Aquitaine breed (beef breed) in the herd (No. of Bd'A)
[7] number of animals of Hereford breed (beef breed) in the herd (No. of Hf)
[8] percentage of animals of beef breeds in the herd (% of beef animals)
[9] regions include Estonian counties: southwest – Pärnu, Saare, and Viljandi counties; southeast – Põlva, Tartu, Valga, and Võru counties; northeast – Ida-Viru, Jõgeva, Järva, and Lääne-Viru counties; and northwest – Harju, Hiiu, Lääne, and Rapla counties.
[10] season of sample collection (Season)
