# Supplementary material for: Coxiella burnetii Seroprevalence and Associated Risk Factors in Cattle, Sheep, and Goats in Estonia
Source: Microorganisms. 2023 Mar 23;11(4):819. doi: 10.3390/microorganisms11040819 (PMC10142450; doi:10.3390/microorganisms11040819)
Supplement: Supplementary file 1 [file microorganisms-11-00819-s001.zip › Table S3.pdf]

Table S3. Univariable regression analysis results to predict *Coxiella burnetii* seroprevalence in bulk tank milk samples from volunteer Estonian dairy cattle herds

Variable	n/N [11]	Prevalence (95% CI [12])	Odds Ratio (95% CI)	p-value
No. of animals [13]			1.002 (1.00–1.003)	0.009
Herd size				
Small (<101 animals)	2/20	10.00 (2.79–30.10)	1	
Medium (101–300 animals)	7/21	33.33 (17.19–54.63)	4.50 (0.81–25.12)	0.085
Large (>300 animals)	16/31	51.61 (34.84–68.03)	9.60 (1.90–48.59)	0.005
Region [14]				
Southwest	8/25	32.00 (17.21–51.59)	1	
Southeast	3/13	23.08 (8.18–50.26)	0.64 (0.14–2.97)	0.567
Northeast	13/18	72.22 (49.13–87.50)	5.53 (1.46–20.90)	0.012
Northwest	1/16	6.25 (1.11–28.33)	0.14 (0.02–1.27)	0.081
Keeping system (lactating cows)				
Tied	5/36	13.89 (6.08–28.66)	1	
Mixed [15]	3/5	60.00 (23.07–88.24)	9.30 (1.23–70.34)	0.031
Loose	17/31	54.84 (37.77–70.84)	7.53 (2.31–24.50)	0.001
Keeping system (pregnant heifers)				
Tied	10/40	25.00 (14.19–40.19)	1	
Mixed	0/3	0.00 (0.00–56.15)	1.64 (0.13–20.94)	0.702
Loose	15/29	51.72 (34.43–68.61)	2.65 (0.92–7.68)	0.084
Animal shows [16]				
No	17/53	32.08 (21.09–45.48)	1	
Yes	8/19	42.11 (23.14–63.72)	1.69 (0.57–5.06)	0.345
Grazing adult animals				
Not used	7/8	87.50 (52.91–97.76)	1	
Dry animals	10/22	45.45 (26.92–65.34)	0.12 (0.01–1.05)	0.055
All animals	8/42	19.05 (9.98–33.30)	0.03 (0.004–0.31)	0.003
Pastures in contact [17]				
No	19/54	35.19 (23.82–48.52)	1	
Yes	1/11	9.09 (1.62–37.74)	0.19 (0.02–1.55)	0.126
N/A[18]	5/7			
Drinking from waterbody [19]				
No	15/42	35.71 (22.99–50.83)	1	
Yes	5/25	20.00 (8.86–39.13)	0.47 (0.15–1.49)	0.199
N/A	5/5			
Animals at home [20]				
No	8/31	25.81 (13.70–43.25)	1	
Yes	16/40	40.00 (26.35–55.40)	0.54 (0.20–1.51)	0.241
N/A	1/1			

[11] number of herds with *CB* antibody positive result (n) and number of tested herds (N)
[12] 95% confidence intervals (95% CI)
[13] number of registered animals in the herd

[14] location areas include the following Estonian counties: southwest – Pärnu, Saare, and Viljandi counties; southeast – Põlva, Tartu, Valga, and Võru counties; northeast – Ida-Viru, Jõgeva, Järva, and Lääne-Viru counties; and northwest – Harju, Hiiu, Lääne, and Rapla counties.
[15] both loose and tied keeping of cattle used
[16] participation in animal shows
[17] the pastures used by the animals are in contact with the neighbouring farm's pastures (Pastures in contact)
[18] no information was available (N/A)
[19] animals are drinking from natural waterbodies during grazing
[20] farm employees are keeping production animals at home
