# Supplementary figures and images for: Coxiella burnetii Seroprevalence and Associated Risk Factors in Cattle, Sheep, and Goats in Estonia
Source: Microorganisms. 2023 Mar 23;11(4):819. doi: 10.3390/microorganisms11040819 (PMC10142450; doi:10.3390/microorganisms11040819)

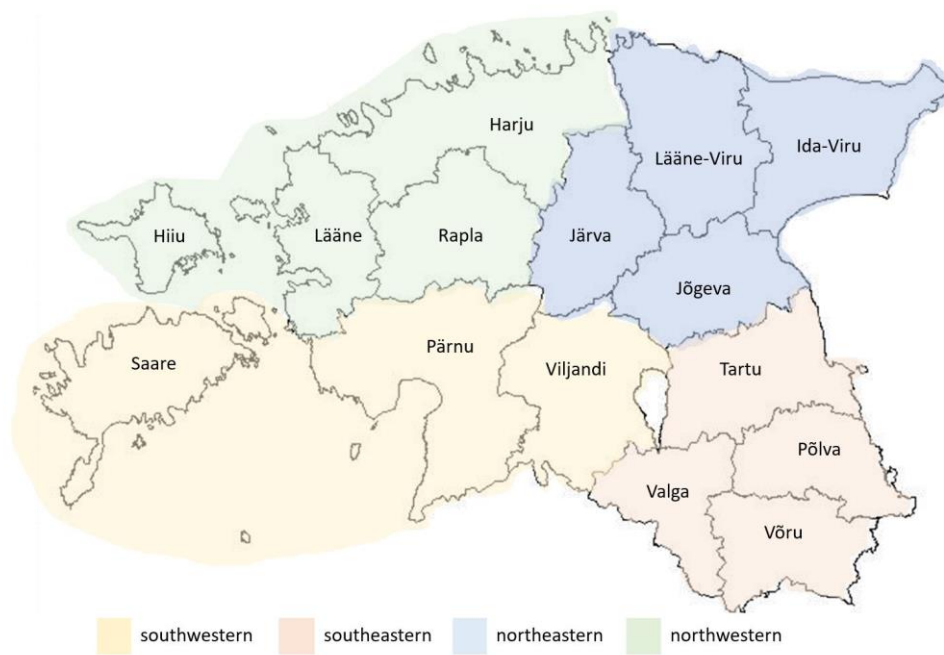

**Figure S2.** Study regions according to Estonian counties

Supplement: Supplementary file 1 [file microorganisms-11-00819-s001.zip › Figure S2.pdf]
